# Electrosprayed Shrimp and Mushroom Nanochitins on Cellulose Tissue for Skin Contact Application

**DOI:** 10.3390/molecules26144374

**Published:** 2021-07-20

**Authors:** Bahareh Azimi, Claudio Ricci, Alessandra Fusco, Lorenzo Zavagna, Stefano Linari, Giovanna Donnarumma, Ahdi Hadrich, Patrizia Cinelli, Maria-Beatrice Coltelli, Serena Danti, Andrea Lazzeri

**Affiliations:** 1 Interuniversity National Consortiums of Materials Science and Technology (INSTM), 50121 Firenze, Italy; bahareh.azimi@ing.unipi.it (B.A.); claudio.ricci@med.unipi.it (C.R.); lorenzo@zavagna.it (L.Z.); patrizia.cinelli@unipi.it (P.C.); 2 Department of Civil and Industrial Engineering, University of Pisa, 56126 Pisa, Italy; andrea.lazzeri@unipi.it; 3 Department of Experimental Medicine, University of Campania “Luigi Vanvitelli”, 80138 Naples, Italy; alessandra.fusco@unicampania.it (A.F.); giovanna.donnarumma@unicampania.it (G.D.); 4 Linari Engineering s.r.l., 56121 Pisa, Italy; stefano.linari@linarisrl.com; 5 Biomass Valorization Platform-Materials, Celabor s.c.r.l., 4650 Chaineux, Belgium; ahdi.hadrich@celabor.be

**Keywords:** surface functionalization, chitin nanofibril, cellulose, skincare, anti-inflammatory, biobased

## Abstract

Cosmetics has recently focused on biobased skin-compatible materials. Materials from natural sources can be used to produce more sustainable skin contact products with enhanced bioactivity. Surface functionalization using natural-based nano/microparticles is thus a subject of study, aimed at better understanding the skin compatibility of many biopolymers also deriving from biowaste. This research investigated electrospray as a method for surface modification of cellulose tissues with chitin nanofibrils (CNs) using two different sources—namely, vegetable (i.e., from fungi), and animal (from crustaceans)—and different solvent systems to obtain a biobased and skin-compatible product. The surface of cellulose tissues was uniformly decorated with electrosprayed CNs. Biological analysis revealed that all treated samples were suitable for skin applications since human dermal keratinocytes (i.e., HaCaT cells) successfully adhered to the processed tissues and were viable after being in contact with released substances in culture media. These results indicate that the use of solvents did not affect the final cytocompatibility due to their effective evaporation during the electrospray process. Such treatments did not also affect the characteristics of cellulose; in addition, they showed promising anti-inflammatory and indirect antimicrobial activity toward dermal keratinocytes in vitro. Specifically, cellulosic substrates decorated with nanochitins from shrimp showed strong immunomodulatory activity by first upregulating then downregulating the pro-inflammatory cytokines, whereas nanochitins from mushrooms displayed an overall anti-inflammatory activity via a slight decrement of the pro-inflammatory cytokines and increment of the anti-inflammatory marker. Electrospray could represent a green method for surface modification of sustainable and biofunctional skincare products.

## 1. Introduction

Chitin is a natural polysaccharide made of N-acetylglucosamine units bound by covalent β-(1→4) linkages and with a structure similar to that of cellulose, thus able to form fibrils and whiskers. Chitin is primarily found in the shells of crustaceans, cuticles of insects, and cell walls of fungi. It is the second most abundant polymerized carbon-based macromolecular material found in nature and can be converted to innovative high-value bio- and eco-compatible materials [1].

Chitin consists of both crystalline and amorphous domains, but the elimination of the amorphous phase results in chitin nanofibrils (CNs), nanocrystals characterized by high bioavailability; thus, they have become an appealing source in green cosmetics [2]. When fibrillated on the nanoscale, chitin loses its pro-inflammatory and allergenic character. Having a backbone structure virtually analogous to that of hyaluronic acid, CN is easily metabolized by the body’s endogenous enzymes, specifically by chitinases and lysozymes present in various human body fluids as well as by some bacterial enzymes present in the colon [2,3]. Controlling the crystalline structure and purity of CNs leads to improvement of its antibacterial, anti-inflammatory, cicatrizing, and anti-aging activity. Hence, CNs are suitable for different applications, mainly in pharmaceutical, biomedical, food, textiles and packaging fields [4,5,6]. By all these properties, in addition to non-toxicity toward living organisms and the environment (i.e., air, water, and soil), chitin can play a role as a biopolymer for sustainable industrial development. Moreover, the antibacterial activity and low immunogenicity of chitin have broadened the aspects of research and development on the structure-function relationship toward biological tissues and activities [1].

Despite the abundance, accessibility, and low cost, the random distribution of acetyl groups in chitin structure, batch-to-batch diversity, and non-accurate characterization of chitin result in a low reproducibility of chitin solubility and eventually in a limitation in product development and access to the market in large volume. Different inorganic acids, bases, and salts have been used for chitin and chitosan dissolution [7].

The electrohydrodynamic atomization technique, simply called electrospray, accrues when a conductive polymer jet separates into very small droplets under the influence of an electrical field [8,9]. The type of solvent is one of the most important parameters in the electrospray technique since, during the flight of droplets to the collector, the primary ones shrink due to the solvent evaporation, which leads to an increase in charge concentration and the breaking of them into smaller offspring [10]. The surface functionalization materials are possible by using the electrospray method.

This study presents the surface patterning of cellulose tissues via electrospray technique using chitin nanofibrils to decorate the surface with functional (e.g., anti-inflammatory, anti-microbial) nano-metric features, for skincare application. Two types of CNs from different sources (i.e., shrimp (sCN) and mushroom (mCN)) were used for the electrospray technique using two solvent systems, which were used to suspend the CNs in liquid media. The effects of CN type and solvent system (i.e., fully and partially water-based) necessary for electrospraying were investigated. In particular, we assessed the cellulosic tissue surface modification via scanning electron microscopy (SEM) and the skin-compatibility using human dermal keratinocytes (HaCaT cells). The expression of a panel of cytokines involved in inflammation and immune response was studied, including the pro-inflammatory interleukins (ILs) IL-1, IL-6, and IL-8; the tumor necrosis factor α (TNF-α); the transforming growth factor β (TGF-β); and the human beta-defensin 2 (HBD-2).

The successful and uniform surface functionalization of cellulose tissue would enable the development of novel biobased products for potential use for skin-related applications, such as skin contact and skincare.

## 2. Results

### 2.1. Morphological Characterization

**Figure 1:**
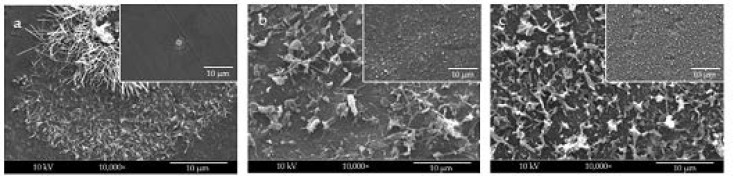
SEM micrographs of electrosprayed sCNs on the aluminum using different solvent systems: (**a**) distilled water, (**b**) distilled water/acetic acid (50/50 *w*/*w*), and (**c**) distilled water/HFIP (60/40 *w*/*w*). Main pictures show zoomed-in (10,000×), while lenses show zoomed-out (1000×) magnifications.

The first step of this study consisted of selecting the most suitable solvent system to electrospray CNs, thus investigating greener systems (i.e., fully and partially water-based). Specifically, Figure 1 shows SEM micrographs of electrosprayed sCNs onto aluminum foil as a back layer and different solvent systems as fluidic carriers. By employing distilled water as a dispersing medium, big droplets containing aggregated CNs were electrosprayed on the surface of aluminum foil, giving rise to non-uniform decoration of the substrate (Figure 1a). However, mixing distilled water with acetic acid (50/50 *w*/*w*) led to the formation of electrosprayed CNs with approximately uniform size and morphology that uniformly covered aluminum foil (Figure 1b). Finally, adding distilled water to hexafluoro-isopropanol (HFIP) (60:40 *w*/*w*) resulted in the smallest size electrosprayed CNs, which uniformly distributed on the surface of the back layer (Figure 1c).

**Figure 2:**
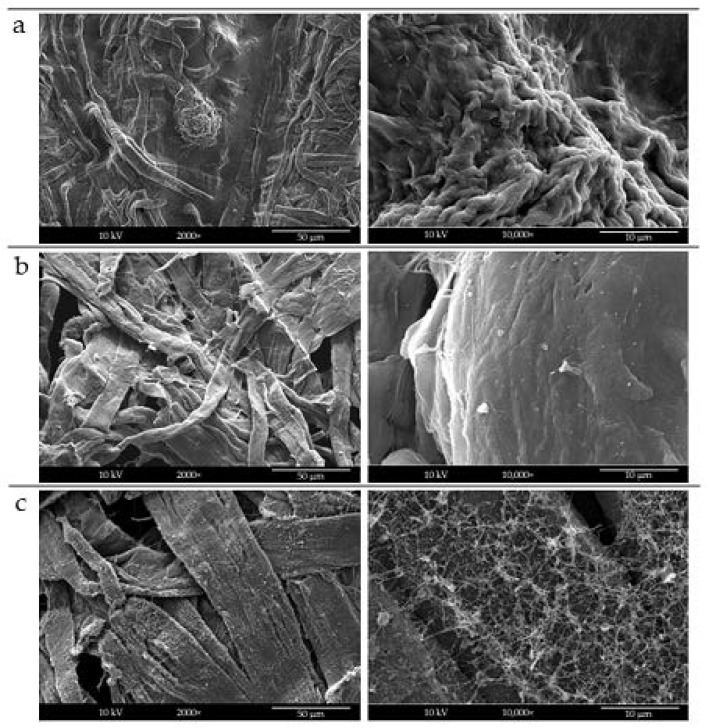
SEM analysis of cellulose tissue decorated with electrosprayed sCNs using different solvent systems: (**a**) distilled water, (**b**) distilled water/acetic acid (50/50 *w*/*w*), and (**c**) distilled water/HFIP (60:40) *w*/*w*). Left column shows zoomed-out (2000×), while right column shows zoomed-in (30,000×) magnifications.

The three solvent systems were then applied to electrospray sCNs onto cellulosic tissues, commonly used for skin contact applications when evaluating whether the deposition mode was affected by a different substrate. Figure 2 shows SEM images of electrosprayed sCNs on the surface of cellulose tissue via different solvents.

**Figure 3:**
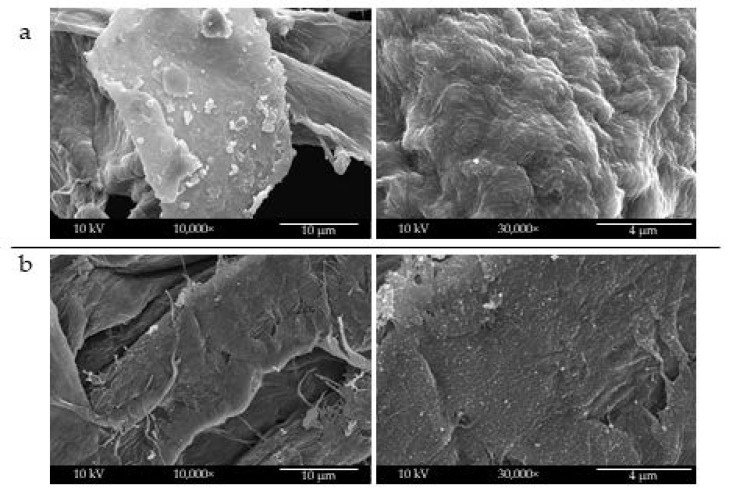
SEM analysis of cellulose tissue decorated with electrosprayed mCNs using different solvent systems: (**a**) distilled water and (**b**) distilled water/acetic acid (50/50 *w*/*w*). Left column shows zoomed-out (10,000×), while right column shows zoomed-in (30,000×) magnifications.

By using distilled water, again only big droplets of sCNs were non-uniformly electrosprayed on the surface of cellulose tissue (Figure 2a). Differently, distilled water/acetic acid (50/50 *w*/*w*) improved the formation of uniform size morphology sCNs that homogeneously deposited onto cellulose tissue (Figure 2b). Interestingly, the solvent system made of distilled water/HFIP (60/40 *w*/*w*), led to the formation of CN nanofiber-like structures that covered the cellulose tissue with a sort of nanostructured texture (Figure 2c). In order to have single CNs to decorate the surface, and the greenest possible solvent systems, we considered only distilled water and distilled water/acetic acid (50/50) to process mCNs. The results obtained by mCNs electrospray are shown in Figure 3. Again, using only distilled water as a solvent gave rise to droplets of aggregated mCNs, and thus their fine distribution was not achieved (Figure 3a). An increased size uniformity and homogeneous distribution of mCNs were obtained by means of distilled water/acetic acid (50/50 *w*/*w*) (Figure 3b).

### 2.2. Chemical Structure Characterization

**Figure 4:**
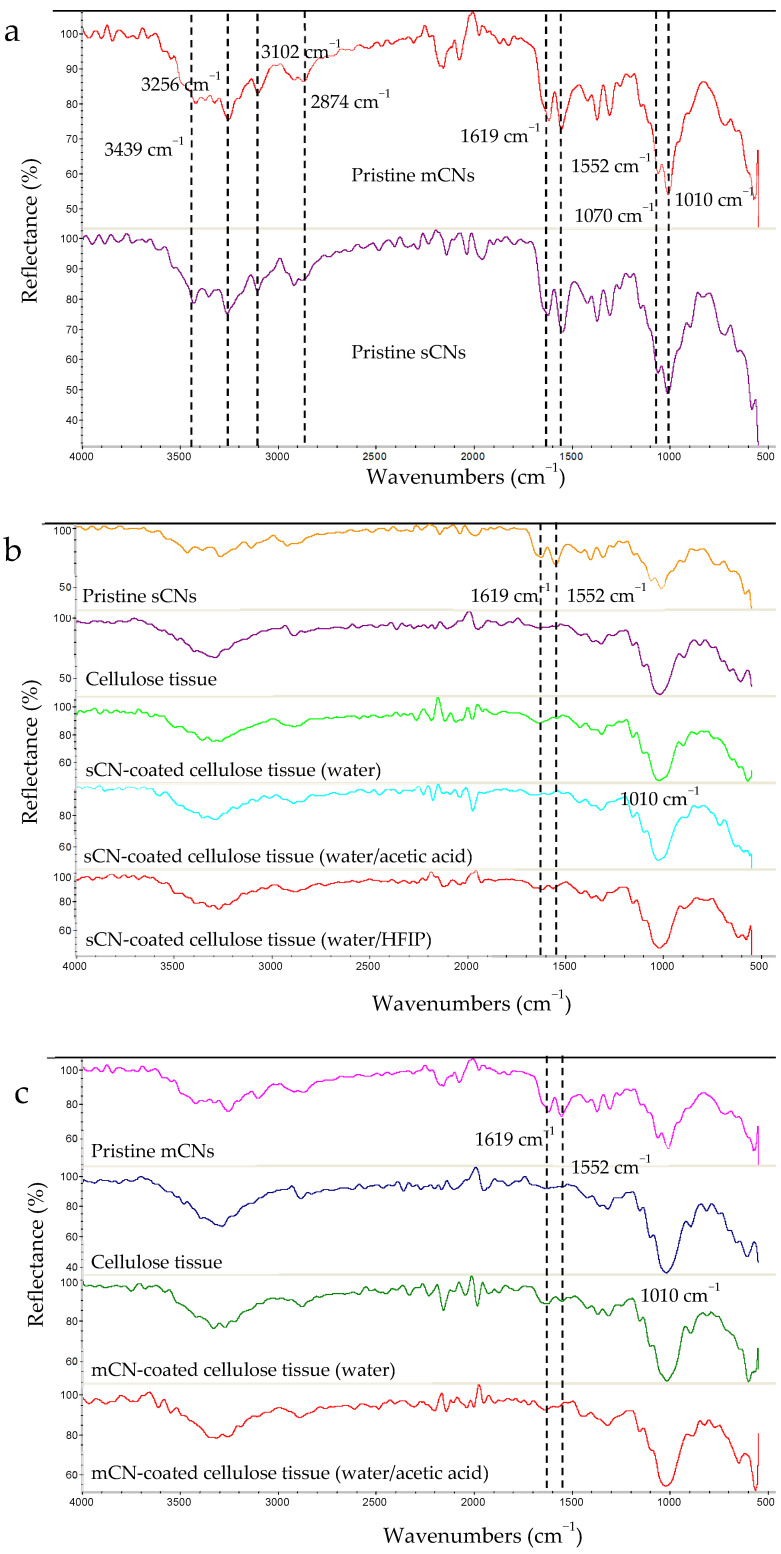
FTIR spectra of: (**a**) dried pristine sCNs and mCNs, (**b**) sCN-coated cellulose tissue using different solvents, and (**c**) mCN-coated cellulose tissue using different solvents.

Figure 4 shows Fourier transform infrared (FTIR) spectra of the dried pristine sCNs and mCNs (Figure 4a), as well as cellulosic tissues coated with sCNs (Figure 4b) and mCNs (Figure 4c) using different solvents. All the characteristic bands of CN—namely, 1010 cm^−1^ and 1070 cm^−1^ typical of C-O stretching, 1552 cm^−1^ attributed to amide II, 1619 cm^−1^ and 1656 cm^−1^ attributed to amide I, 2874 cm^−1^ attributed C-H stretching, 3102 cm^−1^ and 3256 cm^−1^ attributed to N-H stretching of amide and amine groups, and 3439 cm^−1^ attributable to O-H stretching—were revealed in the FTIR spectra of pristine sCNs and mCNs.

The main characteristic bands of CNs (1552 cm^−1^ and 1619 cm^−1^) can be also observed on the spectra of cellulose tissue decorated with electrosprayed sCNs, in particular when water/HFIP (60/40 *w*/*w*) was used as a solvent system (Figure 4b), as well as with electrosprayed mCNs using distilled water as a solvent. Such observations corroborated the presence of CNs on the surface of cellulose tissue. The main characteristic bands of cellulose can also be observed on the surface of cellulose tissue functionalized with electrosprayed CNs. The obtained results indicate that the solvent systems used are safe to keep the CN structure.

### 2.3. Cytotoxicity Evaluation

**Figure 5:**
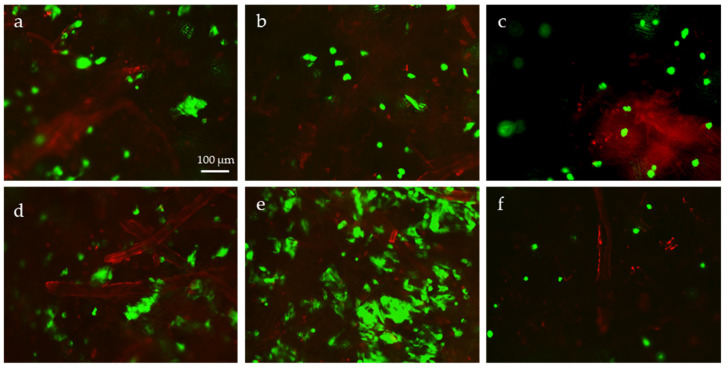
Direct cytotoxicity test: Live/Dead viability test performed on HaCaT cell line seeded on cellulose tissues electrosprayed with: (**a**) sCNs (water); (**b**) sCNs (water/acetic acid); (**c**) sCNs (water/HFlP); (**d**) mCNs (water); (**e**) mCNs (water/acetic acid). (**f**) Pristine cellulose tissue. Viable cells are stained in green, dead cells are stained in red, and the cellulosic tissue shows autofluorescence mainly in the red channel.

**Figure 6:**
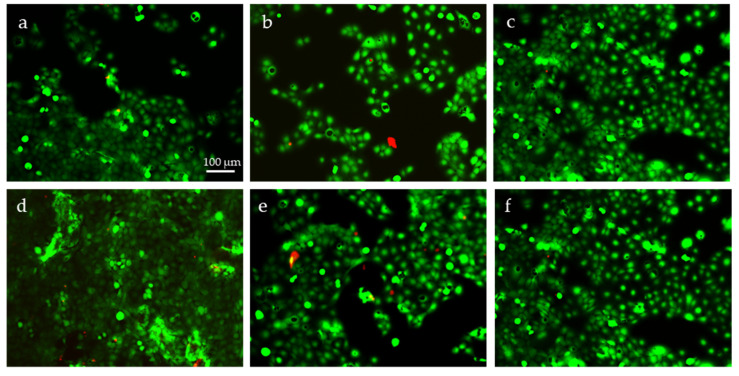
Indirect cytotoxicity test: Live/Dead viability test performed on HaCaT cell line cultured in DMEM previously incubated with cellulose tissues electrosprayed with: (**a**) sCNs (water); (**b**) sCNs (water/acetic acid); (**c**) sCNs (water/HFlP); (**d**) mCNs (water); (**e**) mCNs (water/acetic acid). (**f**) Pristine cellulose tissue. Viable cells are stained in green; dead cells are stained in red.

Both direct (Figure 5) and indirect (Figure 6) cytotoxicity tests revealed that HaCaT cells were viable in all the CN/solvent-treated cellulosic tissues. The cells were viable after being in contact with the tissues (Figure 5), as well as were their eluates in culture media (Figure 6). Only a few dead cells were observed, with no differences between the different samples.

**Figure 7:**
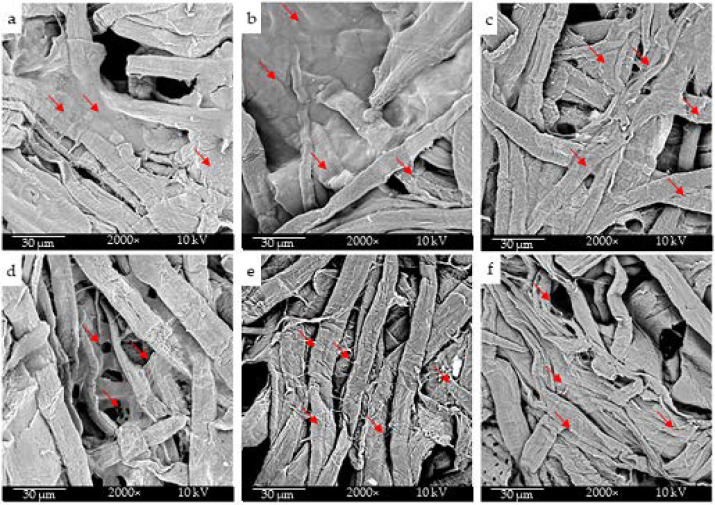
Direct cytotoxicity test: SEM analysis performed on HaCaT cells cultured on cellulose tissues electrosprayed with: (**a**) sCNs (water); (**b**) sCNs (water/acetic acid); (**c**) sCNs (water/HFlP); (**d**) mCNs (water); (**e**) mCNs (water/acetic acid). (**f**) Pristine cellulose tissue. Some dense cell-populated areas are indicated by red arrows. Original magnification 2000×.

SEM analysis (Figure 7) confirmed that the surface of the treated substrates was abundantly covered by a cell layer in all the samples. Overall, the tissue substrate was highly cytocompatible itself, and the presence of electrosprayed CNs did not affect keratinocyte viability. We can conclude that the prepared samples are suitable for skin applications.

### 2.4. Evaluation of Cell Metabolic Activity

An AlamarBlue^®^ test was performed on the HaCaT cells to assess the metabolic activity of different CN-coated cellulose tissues, measured as reduction percentage of AlamarBlue (% AB_red_) dye before performing cytokine expression analysis. The results obtained highlight that after 24 h, HaCaT metabolic activity was higher than 70%, which corroborated good cell viability (Table 1).

### 2.5. Anti-Inflammatory and Immune Response of HaCaT Cells

**Figure 8:**
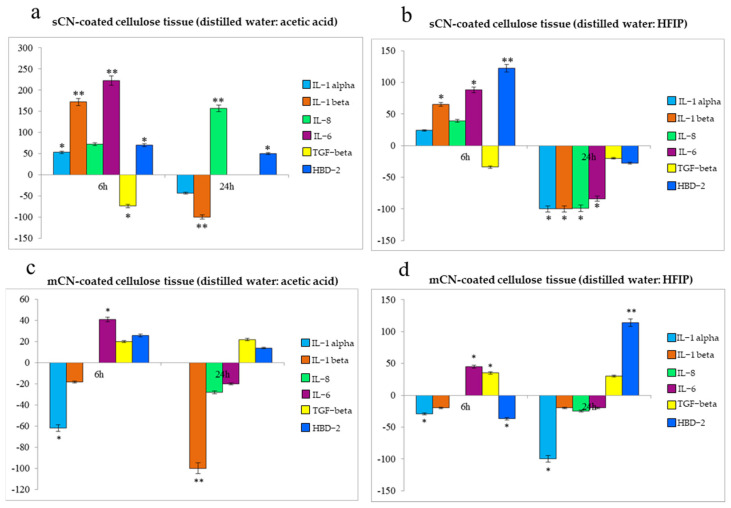
Bar graphs showing the results of quantitative RT-PCR related to different cytokines involved in the inflammatory response of HaCaT cells and HBD-2 produced by HaCaT cells after being exposed to the different CN-coated cellulose tissues for 6 h and 24 h. The results were normalized by the expression in cells treated with pristine cellulosic tissue as a control and thus are given as mRNA expression percentage. Statistically significant differences determined using Student’s *t*-test are indicated by * *p* < 0.05 and ** *p* < 0.001.

HaCaT cells cultured in the presence of the differently treated cellulosic tissues for 6 h and 24 h were analyzed via quantitative real-time polymerase chain reaction (RT-PCR) to determine the expression of a panel of pro- and anti-inflammatory cytokines, along with the expression of an antimicrobial peptide, HDB-2. In order to assess the specific influence played by shrimp or mushroom CNs and the used solvent systems, the results are given as the percentage of mRNA expression with respect to cells cultured on pristine cellulosic tissue (used as control). The outcomes obtained showed a different profile based on the CN sources applied onto the cellulosic substrate (Figure 8). In fact, while the sCNs showed marked early pro-inflammatory and indirect antimicrobial activity (Figure 8a,b), the mCNs displayed a weak pro-inflammatory behavior and a predominant anti-inflammatory activity, with insignificant induction of IL-6 and delayed induction of HBD-2 (Figure 8c,d). Interestingly, sCN-coated cellulose tissues electrosprayed with water/HFIP mixture showed a well-defined downregulation of all the pro-inflammatory cytokines in 24 h (Figure 8b). sCN-coated cellulose tissue using only distilled water as a solvent did not show any difference with respect to pristine cellulose tissue, which confirms the insufficient and inhomogeneous coating. TNF-α, a powerful pro-inflammatory cytokine, was not modulated in any samples.

## 3. Discussion

The skin is the largest organ of the human body that in a lifetime is directly exposed to the sunlight radiation, environmental agents, and chemical pollutants, as well as injuries and other damages; therefore, an appropriate skincare routine is important to maintain health and wellbeing. Due to a frenetic daily life, many people neglect skincare, thus acquiescing to skin stress and immune reactivity. The application of pure active ingredients and anti-inflammatory biomaterials, possibly under the circular economy approach, endowed with intrinsic preventive or even therapeutic properties for skin disorders and aging, is a key topic in modern cosmetology [2,11,12,13].

Cellulose is the most widely available and renewable material on Earth; it is also present as naturally nanofibrous substrates, i.e., bacterial cellulose, imbued with remarkable properties in wound repair, including eardrum perforations [14,15,16,17]. Surface decoration of cellulose tissues with proper functional coatings based on natural biopolymers will allow their cosmetic performance to be improved, thus enabling simple but effective biobased products to effectively replace petrochemical counterparts in the personal care sectors [2]. As an example, CNs, alone and complexed with nanolignin in the form of microcarriers, have shown bioactive features in skin contact and cosmetics, being recently proposed in a novel soluble nonwoven beauty mask based on pullulan [1,6,18]. Electrospray, which is analogous to electrospinning, has aroused interest as an eco-friendly technology: it usually works at room temperature and there are no post-process residues, with a virtual yield of 100% for the electrosprayed active ingredients. In the case of water-based solvent systems, this technique is safe and green, having only electricity as an energy supply [6,19].

In our study, we used the electrospray technique to decorate the surface of cellulose tissue with CNs aimed at improving its antibacterial and anti-inflammatory properties to propose a novel skincare product. We thus investigated shrimp-based and mushroom-based CNs as active ingredients for electrospray and focused on water-based solvent systems. It is well known that a solvent, upon interaction with an electric field, plays a decisive role in the size and the homogeneity of the particles being collected downstream of the electrospray process [20]. Other techniques for surface functionalization in cosmetic and packaging applications, such as spray [21], casting [22], dry powder impregnation [6], multilayer coatings assembled via dipping and spraying [23], or spinning methods [24], have been proposed for modification of different surfaces, including fibrous nonwovens and delicate films using CNs or CN complexes [6,23,24].

CNs are biobased nano compounds that can be considered useful bioactive agents for functionalizing skin contact substrates. In fact, on the nanoscale, chitin is able to modulate the pro- and anti-inflammatory reaction of dermal keratinocytes [1,25]. The application of an easy and efficient method for decorating the biomaterial surfaces with such components and for quality control of the outcomes has been the subject of recent studies [26]. Water-based spray of CNs may affect the cellulose substrate surface by wetting. Searching for environmentally friendly solvents, we thus considered that electrospray could be a valuable method for CN deposition on cellulose tissue surfaces, as it can uniformly functionalize the surface of the substrate without a wetting effect due to the nanodrops being generated in presence of a high voltage field. Different parameters can affect the production of nanoparticles with uniform size and morphology during the electrospray process [27,28,29]. Solution parameters include the solvent type and solution viscosity, and electrospinning parameters include applied voltage, flow rate, and needle tip to collector distance. In fact, a polymer solution used for the electrospray technique should be enough diluted so that a low sufficient viscosity allows the solution to break up into droplets at the same time so as to not be too viscous to form the continuous fibers [8,10]. The volatility of solvents is also important since the electrospray principle is based on solvent evaporation.

Usually, aggregated three-dimensional (3D) chitin networks resulting from high crystallinity, hydrogen bonds, and strong cohesive forces cause the lack of solubility in conventional solvents, which has limited its applications [7]. In our study, the so-called “solvent systems” were selected to electrospray finely dispersed CNs in a liquid means. In this way, the CN properties were better preserved, as chitin was neither dissolved to form a solution (rather CN was in a colloidal suspension state with the solvent systems) nor deacetylated. Therefore, in our intent, the primary role of the solvent system was to allow separation among the CNs, along with an efficient interaction between the CNs and the electric field, which in turn results in smaller size droplets and larger deposited areas. We investigated the effect of CN source and solvent system types on the decoration of cellulose tissues and their bioactive properties for skin contact applications. Using distilled water as a dispersion agent resulted in the formation of large size droplets containing aggregated CNs for both shrimp and mushroom types. This phenomenon occurred when electrospray was performed using both aluminum and cellulose tissue as collecting surfaces. Using water/acetic acid as a solvent led to the deposition of CNs with uniform size and morphology, in particular when mCNs were employed as a chitin source. The observed differences can be due to the improved nano-suspension of CNs in an acidic solution [21]. In the acidic system, the protonation of -NH_2_ groups present on the surface of CNs occurs, leading to the formation of positive charges at the surface. The repulsions between nanofibrils counteract coalescence phenomena, thus favoring a homogenous deposition by electrospray.

The improved behavior obtained by adding 50% *w*/*w* glacial acetic acid can be explained by the change in surface tension, which enables better interaction with the electric field. It has been reported that to electrospinning the chitosan-a partially deacetylated form of chitin, the use of acetic acid/water solvents at higher ratios (10% up to 90%) improved the fiber formation by decreasing the surface tension, which is considered a key parameter to switch from particles into beads or fibers. In this study, 50% acetic acid still was in the particle formation window [30].

As a further example of organic solvent miscible in water, we tested HFIP, a polar fluoroalcohol with strong hydrogen bonding properties, which has an acidic behavior. HFIP is able to solubilize mostly insoluble polymers, peptides, and β-sheet protein aggregates. By using the mixture of distilled water/HFIP (60/40 *w*/*w*%), continuous chitin nanofiber webs with a homogeneous morphology and a quite uniform diameter were formed on the surface of the cellulose tissue. This phenomenon has been described by dissolving chitin in pure HFIP, as an in vitro self-assembly (i.e., bottom-up) of biogenic chitin nanofibers initiated by solvent evaporation [31]. The authors did not observe deacetylation under the mild conditions applied, and the crystalline structure of chitin was maintained. Apparently, the application of HFIP as a co-solvent in an equal concentration to water led to the formation of a CN solution with a higher viscosity than the solution produced with distilled water or diluted acetic acid. Such a viscous solution led to the formation of CN nanofibers instead of CNs. Min et al. investigated the variation in viscosity with the concentration of chitin in HFIP [32]. They demonstrated that at lower concentrations, fibers with a large number of beads were formed while increasing concentration led to the significant chain entanglements and subsequent increase of the viscosity, which ultimately formed the continuous fibers of chitin.

We showed that electrospray is a safe technology to decorate cellulose tissue since no significant difference was observed in the chemical structure of cellulose tissue after its surface modifications and in the main characteristic bands of CN observed on the spectrum of cellulose tissue functionalized with CN from different sources. Chitins may have different acetylation depending on the source type, such as fungi, insects, crustaceans, mollusks, and the extraction processes, which may determine variations in their biological activity [7]. The search for animal-free cosmetic and skincare products is looking specifically at vegetable sources of chitins, such as mushrooms, whereas the biobased industries are particularly interested in chitin as a biowaste, such as crustaceans. Therefore, the diverse chitin source can comply with the specific needs of the market, and an investigation about the specific biological properties of different CNs could enable the best choice for each final application. After both sCNs and mCNs were electrosprayed via diverse water-based solvent systems upon cellulosic tissues, the functionalized paper was cytocompatible with HaCaT cells chosen as an epidermal skin model. Previous studies performed on free sCNs and electrosprayed sCNs onto polyhydroxyalkanoate fiber meshes corroborated the good interaction of sCNs with HaCaT cells [1,9]. There are many recent studies that investigated cellulose as a scaffold, also reporting excellent properties of paper in cell cultures in vitro and cell migration in vivo [14,33,34,35,36]. In our results, the CN functionalized cellulosic tissue was highly cytocompatible in vitro using a model of human dermal keratinocytes under indirect and direct testing modes. 

Independent of the solvent systems applied for CN dispersion, viable HaCaT cells were detected in all the samples and conditions used. Apparently, a few dead cells were observed in the indirect cytotoxicity tests in both samples processed using water/acetic acid to electrospray the CNs, in which also the metabolic activity was slightly lower than in the other samples. Even though this fact could be a consequence of some acidic residue of acetic acid, the direct cytocompatibility tests corroborated the very good cytocompatibility of these samples. Acetic acid is a weak organic acid of natural origin; thus, its environmental impact is lower than that of the other acids tested, i.e., HFIP. In the electrospinning process, the solvents are trapped in a filter, which must be disposed or possibly regenerated with solvent recovery [37]. In the case of a solvent mixture, such as water/solvent, the separation process and cost must be evaluated. Moreover, the use of HFIP led to the obtainment of self-assembled chitin nanofibers instead of separate CNs, so the mixture of water/acetic acid could be better suitable for a circular economy approach [2].

Finally, small differences in biological activity were detected between sCNs and mCNs. Specifically, the cellulose substrate decorated with sCNs showed strong immunomodulatory activity; conversely, using mCNs had an overall anti-inflammatory activity. The different reactions of HaCaT cells to the substrates covered by different CNs could be explained by the diverse immune recognition, which is also affected by size and shape, as well as source and purification methods [38]. It has been demonstrated that chitin lower than 0.2 µm loses immunogenicity; however, aggregated CNs may be perceived differently from the cells. Other possible concurring factors are physico-chemical treatments used to purify chitin, which can alter the chitin structure, thus its recognition by the immune cells [38]. Therefore, all these aspects must be taken into account when developing a cosmetic product using CNs.

## 4. Materials and Methods

### 4.1. Materials

Both sCNs (water solution, 1.5%) and mCNs (Glentham, Mushroom-based) (water solution, 1.5%) were supplied by Celabor (Mouscron, Belgium). Cellulose tissue used as a substrate was kindly provided by LUCENSE (Lucca, Italy). Acetic acid (code: 33209) and hexafluoro-2-propanol (HFIP) (code: 105228) were bought from Sigma-Aldrich (Milan, Italy). HaCaT cell line was obtained from CLS–Cell Lines Service, Eppelheim, Germany. Absolute ethanol was supplied by VWR (by Avantor, Radnor, PA, USA). Formalin was purchased from BioOptica (Milan, Italy). Fetal bovine serum (FBS) and Live/Dead kit (propidium iodide and calcein, AM, USA) were bought from Gibco (by Life Technologies, ThermoFisher Scientific, Waltham, MA, USA). Dulbecco’s Modified Essential Medium (D-MEM), l-Glutamine, penicillin, streptomycin, MgCl_2_, LiCl, LC Fast Start DNA Master SYBR Green Kit, and Tri Reagent^®^ were purchased from Sigma-Aldrich (Merck KGaA, Darmstadt, Germany). Phosphate buffered saline (PBS) was supplied by Lonza (Basel, Switzerland).

### 4.2. Preparation of CN Solutions and Electrospray Protocols

CN was used at 0.52 *w*% in all applied solvents. Distilled water, distilled water/acetic acid (50:50 *w*/*w*), and distilled water/HFIP (60:40) *w*/*w*) were used as solvents. Each solution was magnetically stirred for 3 h until it appeared uniform. Each solution was electrosprayed using an electrospinning bench apparatus (Linari Engineering s.r.l., Pisa, Italy) with a distance of 7 cm from the positively charged needle tip to the grounded aluminum static collector at 17 kV at a flow rate of 0.136 mL/h for 30 min.

In the following step, cellulose tissue was connected to the static aluminum collector, and for each CN solution, the same electrospray parameters were used for 3 h to electrospray CN on the surface of cellulose tissues.

### 4.3. Morphological Characterization of CNs

Morphological analysis of the samples was performed using field emission electron scanning microscopy (FE-SEM) with FEI FEG-Quanta 450 instrument (Field Electron and Ion Company, Hillsboro, OR, USA) and inverted optical microscope (Nikon Ti, Nikon Instruments, Amsterdam, The Netherlands). The samples were sputtered with gold or platinum for analysis. ImageJ software (version 1.52 t) was used to evaluate the size of nanofibrils and fibers. An average of 50 measurements was reported for each sample.

### 4.4. Chemical Structure Characterization

Fourier transform infrared spectroscopy (FTIR) was pursued using a Nicolet T380 instrument (Thermo Scientific, Waltham, MA, USA) equipped with a Smart ITX ATR attachment with a diamond plate, which was employed for chemical structure characterization of both solid chitin-based substances and cellulose tissue.

### 4.5. Cytotoxicity Evaluation with Epidermal Cells

Human keratinocytes HaCaT cells were employed to investigate the cytocompatibility of the different coatings of the cellulosic substrates using both a direct and an indirect cytotoxicity evaluation method. The cells were expanded in 25 cm^2^ tissue culture flasks using DMEM supplemented with 10% FBS, 1% l-glutamine, and 1% penicillin-streptomycin until semi-confluence was reached. Briefly, 4 coated samples + 4 uncoated controls were cut into 1 cm^2^ square, sterilized by immersion in absolute ethanol 12 h, washed in PBS, and seeded with 2.5 × 10^4^ cells/sample.

A direct cytotoxicity test was performed by adding 20 µL of a HaCaT suspension in a culture medium containing 1∙10^5^ cells on the top surface of cellulosic tissues previously layered onto 24-well plates (*n* = 2). The samples were placed to rest in a humidified incubator (95% air/5% CO_2_) at 37 °C for 30 min to allow cell adhesion. Then, 1.5 mL of culture medium was added to each sample and the culture was continued for 36 h. To perform a Live/Dead assay, the cellularized samples were added with sterile PBS, containing the fluorescent dyes according to the manufacturer’s protocol, and were observed under an inverted fluorescence microscope equipped with a camera (Nikon-Ti, Tokyo, Japan).

At the endpoint, the samples were fixed in 10% formalin for 10 min, rinsed in distilled water, and dried in a vacuumed oven set at 37 °C. The specimens were mounted on aluminum stumps, sputter-coated via an argon gas carrier for 15 s (Coater SC7620, Quorum Technologies Ltd., West Sussex, UK), and observed by SEM (Phenom Pro, Thermo Scientific). 

The indirect cytotoxicity assay was carried out by plating HaCaT cells into 24-well plates at 1∙10^5^ cells/well, including wells for untreated controls, and let to adhere overnight (*n* = 2). Samples of cellulosic tissues with the coatings, including uncoated controls, previously sterilized as above, were placed in tubes containing culture medium (2 cm^2^/tube/2.5 mL culture medium) for 18 h. The subsequent day, the culture media in the cell samples were replaced with the conditioned media. To perform Live/Dead assay, 36 h after seeding, the media were removed from the wells, and the cells were added with sterile PBS containing the fluorescent dyes according to the manufacturer’s protocol and were observed under an inverted fluorescence microscope equipped with a camera (Nikon-Ti, Tokyo, Japan).

### 4.6. Cell Metabolic Activity Evaluation

The HaCat cells, cultured as described above and seeded in 12-well plates until 80% of confluence, were incubated for 24 h with the sterile CN/cellulose tissues. At the end of this time, resazurine was added to the concentration of 0.5 mg/mL and incubated for 4 h. At the endpoint, the AlamarBlue test was performed following the manufacturer’s protocol. Briefly, AlamarBlue incorporates a redox indicator that changes color according to cell metabolic activity. The supernatants were read with a spectrophotometer using a double wavelength reading at 570 nm and 600 nm. Finally, the reduced percentage of the dye (% AB_RED_) was calculated by correlating the absorbance values and the molar extinction coefficients of the dye at the selected wavelength following the protocol provided by the manufacturer. The equation applied is shown below, in which: λ = absorbance, *s* = sample, and *c* = control. The results obtained are expressed as % AB_red_, which is related to metabolically active cells.

(1) $${\%\;\mathrm{AB}}_{\mathrm{red}}=100\times \frac{\left(117,216\times \lambda_{s\left(570\;\mathrm{nm}\right)}-\;80,586\times \lambda_{s\left(600\;\mathrm{nm}\right)}\right)}{\left(155,677\times \lambda_{c\left(600\;\mathrm{nm}\right)}-\;14,652\times \lambda_{c\left(570\;\mathrm{nm}\right)}\right)}$$

### 4.7. Immune Response by Epidermal Cells

The immunomodulatory properties of decorated cellulose substrates were assayed using HaCaT cells. The films, sterilized overnight in EtOH and rinsed 3 times with PBS, were placed on the bottom of 6-well plates, then HaCaT cells were plated on them and incubated for 6 h and 24 h with the cellulosic tissues (*n* = 3). At the endpoint, the mRNA was extracted from the cells, and the levels of expression of the proinflammatory cytokines IL-8, IL-6, IL-1 β, IL-1 α, and TNF-α anti-inflammatory cytokine, TGF-β, and antimicrobial peptide HBD-2 were evaluated by real-time PCR. Briefly, the total RNA was isolated with TRizol, and 1 µm of RNA was reverse-transcribed into complementary DNA (cDNA) using random hexamer primers at 42 °C for 45 min according to the manufacturer’s instructions. Real-time polymerase chain reaction (PCR) was carried out with the LC Fast Start DNA Master SYBR Green kit using 2 µL of cDNA, corresponding to 10 ng of total RNA in a 20 µL final volume, 3 mM MgCl_2_, and 0.5 µM sense and antisense primers (Table 2).

### 4.8. Statistical Analysis

Each marker expression level was compared against its untreated control using Student’s *t*-test. Probability (*p*) values < 0.05 were considered as statistically significant differences.

## 5. Conclusions

An easy and efficient method was set up to uniformly decorate the surface of cellulose tissue via electrospray of CNs extracted from different sources, i.e., shrimp- and mushroom-derived. Among different water-based solvent systems tested for CN dispersion, a mixture of water and acetic acid (50/50%) was the most effective to have the cellulose tissue decorated with low aggregated CNs. We performed direct and indirect cytotoxicity tests to evaluate the compatibility in vitro with HaCaT cells, which successfully adhered to the tissue and were highly viable in culture media conditioned with the material supernatants. The use of solvents did not affect the final cytocompatibility as a result of their effective evaporation during the electrospray process. Such completely biobased functional tissues possessed promising anti-inflammatory and indirect antimicrobial activity, even though slight differences could be observed according to the diverse CN source/solvent system. On the whole, the green method for surface modification of sustainable and biofunctional skincare products could allow effective treatment of irritated skin by giving novel alternatives to the field of green cosmetics.

## Figures and Tables

**Table 1:** Average metabolic activity given as AlamarBlue reduction percentage (% AB_red_) performed at 24 h performed on HaCaT cells in presence of cellulose tissue coated via different CN/solvent suspensions (*n* = 2).

Cellulose Tissue Sample	Electrospray Solvent(s)	% AB_RED_
sCN-coated	distilled water	73%
sCN-coated	distilled water/acetic acid	79%
sCN-coated	distilled water/HFIP	98%
mCN-coated	distilled water	95%
mCN-coated	distilled water/acetic acid	88%
Pristine	none	71%

**Table 2:** Analyzed genes involved in the immune response of HaCaT cells exposed to CN-coated cellulosic tissues.

Gene	Primer Sequence (Forward, Reverse)	Conditions	Base Pairs
IL-1α	5′-CATGTCAAATTTCACTGCTTCATCC-3′ 5′-GTCTCTGAATCAGAAATCCTTCTATC-3′	5 s at 95 °C, 8 s at 55 °C, 1 s at 72 °C for 45 cycles	421
IL-1β	5′-GCATCCAGCTACGAATCTCC-3′ 5′-CCACATTCAGCACAGGACTC-3′	5 s at 95 °C, 14 s at 58 °C, 28 s at 72 °C for 40 cycles	708
TNF-α	5′-CAGAGGGAAGAGTTCCCCAG -3′ 5′-CCTTGGTCTGGTAGGAGACG-3′	5 s at 95 °C, 6 s at 57 °C, 13 s at 72 °C for 40 cycles	324
IL-6	5′-ATGAACTCCTTCTCCACAAGCGC-3′ 5′-GAAGAGCCCTCAGGCTGGACTG-3′	5 s at 95 °C, 13 s at 56 °C, 25 s at 72 °C for 40 cycles	628
IL-8	5-ATGACTTCCAAGCTGGCCGTG-3′ 5-TGAATTCTCAGCCCTCTTCAAAAACTTCTC-3′	5 s at 94 °C, 6 s at 55 °C, 12 s at 72 °C for 40 cycles	297
TGF-β	5′-CCGACTACTACGCCAAGGAGGTCAC-3′ 5′-AGGCCGGTTCATGCCATGAATGGTG-3′	5 s at 94 °C, 9 s at 60 °C, 18 s at 72 °C for 40 cycles	439
HBD-2	5′-GGATCCATGGGTATAGGCGATCCTGTTA-3′ 5′-AAGCTTCTCTGATGAGGGAGCCCTTTCT-3′	5 s at 94 °C, 6 s at 63 °C, 10 s at 72 °C for 50 cycles	198

## Data Availability

The data presented in this study are available on request from the corresponding authors.

## Sample Availability

Samples of the compounds are available on request from corresponding authors.
